# Supplementary material for: Membrane Tethering Potency of Rab-Family Small GTPases Is Defined by the C-Terminal Hypervariable Regions
Source: Front Cell Dev Biol. 2020 Sep 30;8:577342. doi: 10.3389/fcell.2020.577342 (PMC7554592; doi:10.3389/fcell.2020.577342)
Supplement: Supplementary Figure 1 — Dynamic light scattering measurements of synthetic liposomes used in the present reconstitution studies. (A,B) Histograms of size distributions of typical liposome preparations, the Rh-labeled 200-nm complete liposomes containing PC, PE, PI, PS, and cholesterol (A) and the Rh-labeled 200-nm PC/PE liposomes containing PC and PE (B), measured by dynamic light scattering. [file Presentation_1.pdf]

Supplemental Figures

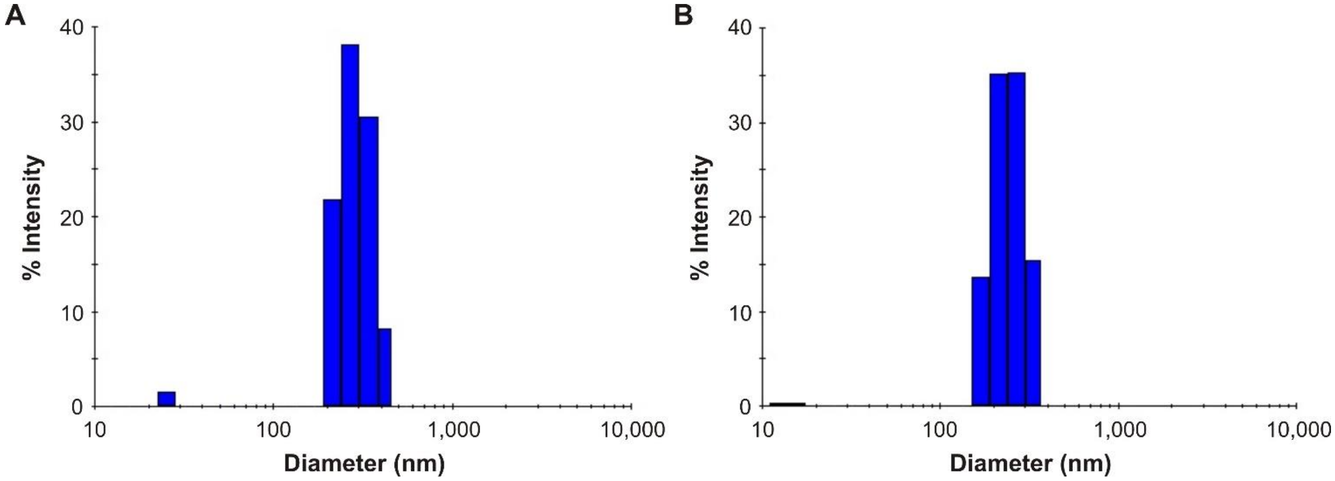

Figure S1. Dynamic light scattering measurements of synthetic liposomes used in the present reconstitution studies. (**A**-**B**) Histograms of size distributions of typical liposome preparations, the Rh-labeled 200-nm complete liposomes containing PC, PE, PI, PS, and cholesterol (**A**) and the Rh-labeled 200-nm PC/PE liposomes containing PC and PE (**B**), measured by dynamic light scattering.

Supplemental Figures

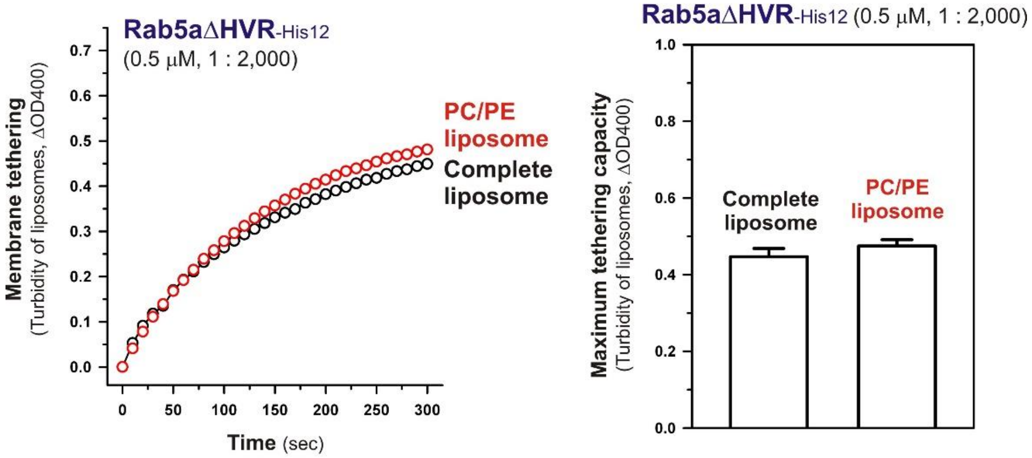

Figure S2. Liposome turbidity assays for Rab5aΔHVR-mediated membrane tethering with the complete liposomes and PC/PE liposomes, testing at the lower Rab-to-lipid molar ratios of 1:2,000. Rab5aΔHVR-His12 proteins (final 0.5 μM) were mixed with the complete liposomes or PC/PE liposomes (200-nm diameter; final 1 mM lipids) and then assayed for the turbidity changes (left panel) as in Figure 7C, but at the Rab-to-lipid molar ratios of 1:2,000. The maximum tethering capacities of Rab5aΔHVR (right panel) were determined from the kinetic data, as in Figure 7C. Error bars, SD.

Supplemental Figures

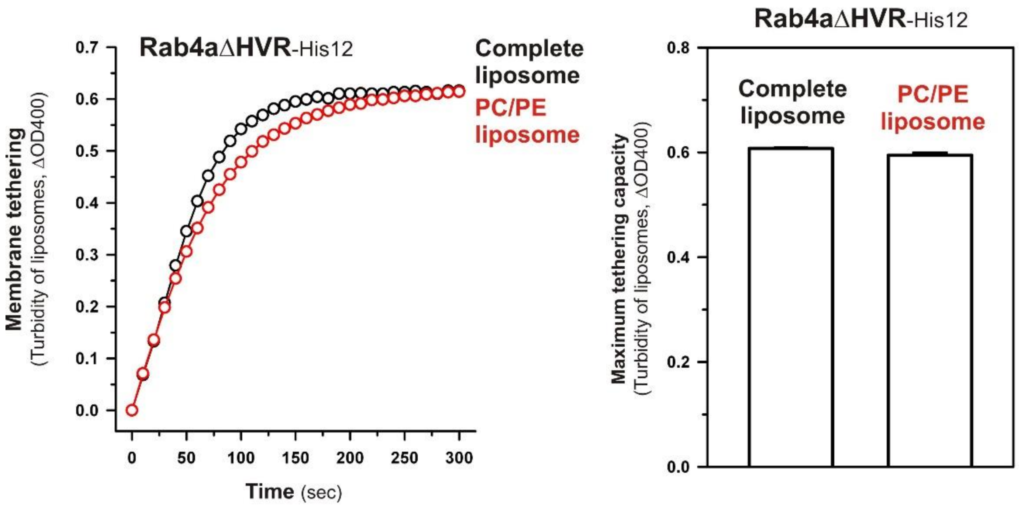

Figure S3. Liposome turbidity assays for Rab4aΔHVR-mediated membrane tethering with the complete liposomes and PC/PE liposomes. Rab4aΔHVR-His12 proteins (final 1 μM) were mixed with the complete liposomes or PC/PE liposomes (200-nm diameter; final 1 mM lipids) and then assayed for the turbidity changes (left panel), as in Figure 7 and Figure 9. The maximum tethering capacities of Rab4aΔHVR (right panel) were determined from the kinetic data, as in Figure 7 and Figure 9. Error bars, SD.
